# Whole-Genome Sequencing of Retinoblastoma Reveals the Diversity of Rearrangements Disrupting *RB1* and Uncovers a Treatment-Related Mutational Signature

**DOI:** 10.3390/cancers13040754

**Published:** 2021-02-11

**Authors:** Helen R. Davies, Kevin D. Broad, Zerrin Onadim, Elizabeth A. Price, Xueqing Zou, Ibrahim Sheriff, Esin Kotiloğlu Karaa, Irene Scheimberg, M. Ashwin Reddy, Mandeep S. Sagoo, Shin-ichi Ohnuma, Serena Nik-Zainal

**Affiliations:** 1 Academic Department of Medical Genetics, University of Cambridge, Addenbrooke’s Treatment Centre, Cambridge Biomedical Campus, Cambridge CB2 0QQ, UK; hd10003@hutchison-mrc.cam.ac.uk (H.R.D.); xz388@MRC-CU.cam.ac.uk (X.Z.); 2 MRC Cancer Unit, Hutchison/MRC Research Centre, Cambridge Biomedical Campus, University of Cambridge, Cambridge CB2 0XZ, UK; 3 Institute of Ophthalmology, University College London, London EC1V 9EL, UK; kevinbroad@btinternet.com; 4 Retinoblastoma Genetic Screening Unit, The Royal London Hospital, Barts Health NHS Trust, London E1 1FR, UK; z.onadim@nhs.net (Z.O.); elizabeth.price11@nhs.net (E.A.P.); 5 Retinoblastoma Service, Royal London Hospital, Barts Health Trust, London E1 1FR, UK; ibrahim.sheriff@nhs.net (I.S.); ashwin.reddy4@nhs.net (M.A.R.); 6 Pathology Department, Royal London Hospital, Barts Health NHS Trust, London E1 1FR, UK; e.karaa@nhs.net (E.K.K.); irenescheimberg@gmail.com (I.S.); 7 NIHR Biomedical Research Centre for Ophthalmology, Moorfields Eye Hospital, Institute of Ophthalmology, University College London, London EC1V 2PD, UK

**Keywords:** retinoblastoma, whole-genome sequencing, structural rearrangement, mutational signature

## Abstract

**Simple Summary:**

Retinoblastoma, a childhood cancer of the eye, is thought to be caused by inactivating mutations of both copies of the *RB1* gene. The majority of *RB1* mutations can be detected by clinical screening. However, retinoblastoma cases exist where mutations in *RB1* have not been detected. We used whole-genome sequencing to investigate the landscape of mutations in a cohort of sporadic retinoblastomas, including cases where mutations in both copies of *RB1* had not been previously identified. We looked for mutations in cancer driver genes and revealed a wide variety of structural rearrangements disrupting *RB1*. In addition, we investigated mutation burden and specific mutation patterns (mutational signatures), uncovering a treatment-related mutational signature in a tumour exposed to chemotherapy. The power of whole-genome sequencing to identify *RB1* mutations of all mutation types can have significant relevance to the clinical management of retinoblastoma patients and genetic counselling of their families.

**Abstract:**

The development of retinoblastoma is thought to require pathological genetic changes in both alleles of the *RB1* gene. However, cases exist where *RB1* mutations are undetectable, suggesting alternative pathways to malignancy. We used whole-genome sequencing (WGS) and transcriptomics to investigate the landscape of sporadic retinoblastomas derived from twenty patients, sought *RB1* and other driver mutations and investigated mutational signatures. At least one *RB1* mutation was identified in all retinoblastomas, including new mutations in addition to those previously identified by clinical screening. Ten tumours carried structural rearrangements involving *RB1* ranging from relatively simple to extremely complex rearrangement patterns, including a chromothripsis-like pattern in one tumour. Bilateral tumours obtained from one patient harboured conserved germline but divergent somatic *RB1* mutations, indicating independent evolution. Mutational signature analysis showed predominance of signatures associated with cell division, an absence of ultraviolet-related DNA damage and a profound platinum-related mutational signature in a chemotherapy-exposed tumour. Most *RB1* mutations are identifiable by clinical screening. However, the increased resolution and ability to detect otherwise elusive rearrangements by WGS have important repercussions on clinical management and advice on recurrence risks.

## 1. Introduction

The retinoblastoma (Rb) is the most common primary intraocular cancer of childhood accounting for approximately 2% of all childhood cancers [1], causing significant long-term sequelae. Loss-of-function mutations in RB transcriptional co-repressor 1 (*RB1*) are causally implicated in Rb development. *RB1* was the first tumour suppressor gene to be isolated, in contrast to activating oncogenes, which were characterised earlier [2,3]. In humans, *RB1* is located on chromosome 13q14.2, spans approximately 180 Kb and contains 27 exons encoding a 928 amino acid nucleophosphoprotein, known as pRB [4]. 

pRB is a multifunctional protein involved in a variety of processes regulating cell proliferation at multiple levels including apoptosis, histone methylation and chromatin remodelling [5,6,7,8]. Loss of function of pRB in the retina is thought to lead to dysregulation of these events, resulting in uncontrolled cell proliferation and chromosomal instability. 

Rb patients present with a wide spectrum of *RB1* mutations. The prevailing view is that mutations in both alleles of the *RB1* gene are required to enable tumourigenesis [9]. Heritable predisposition is present in ~45% of cases and is transmitted in an autosomal dominant manner. Typically, penetrance is almost complete (>90%) and is characterised by the development of tumours in both eyes (bilateral retinoblastomas) in most cases. Germline *RB1* mutation carriers are also at an increased lifetime risk of non-ocular tumours [10]. In 55% of cases, somatically-acquired *RB1* mutations underpin tumourigenesis, are unilateral and do not incur predisposition to develop tumours at other sites [11,12,13,14]. 

Although Knudson’s “two-hits hypothesis” suggests that a loss of function of both *RB1* alleles is required to initiate retinoblastoma [15], using standard clinical screening techniques, in 3–4% of tumours, only one *RB1* mutation can be identified [9,16]. Indeed, in approximately 2% of tumours, *RB1* mutations are elusive [17,18]. This suggests that undetected *RB1* mutations possibly involving translocations, intronic mutations or novel promoter mutations may be harboured by these tumours, or the development of some retinoblastoma tumours may be driven through an *RB1*-independent mechanism [17,19]. Two papers recently highlighted the importance of other potential driver mutations including focal *MYCN* amplification, first reported in 1–2 % of tumours with no detectable *RB1* mutations [17,19]. Focal amplifications of the orthodenticle homeobox 2 gene (*OTX2*) were observed in 3% and focal deletions of the BCL6 Corepressor (*BCOR*) observed in 4% of the same cohort of retinoblastoma tumours [19]. Truncating point mutations in *BCOR* have been also reported in 10–17% of Rb [18,20,21,22]. In addition, a number of studies have identified consistent large-scale copy number changes including gains of 1q, 2p and 6p and losses of 16q [19,20,23]. The contribution of structural mutational mechanisms to *RB1* disruption has also been reported [19]. 

To date, the majority of studies investigating genomic aberrations in Rb have focused on copy number aberrations [23], whole-exome sequencing [20] or targeted gene panels [18,22]. Very limited numbers of whole-genome sequencing (WGS) experiments have been performed. WGS of 4 Rb tumours was performed by Zang et al., with *RB1* being the only mutated known cancer gene identified [21]. The data from the same 4 WGS were also included in a combined analysis of 547 paediatric tumours from 24 cancer types [24]. In a separate study by McEvoy et al., WGS of 10 tumours identified 3 cases with chromothripsis at the *RB1* locus [19]. In this study, we performed WGS on a total of 21 retinoblastomas from 20 individuals with no previous family history (3 with bilateral and 17 unilateral tumours). In addition, transcriptomic profiling was performed on a subset of tumours. The Rb tumours comprised two cohorts: Cohort 1, made up of nine tumours, where two distinct *RB1* mutations were previously identified in each tumour; Cohort 2 comprised ten tumours, where seven had only one *RB1* mutation, and in three, *RB1* mutations remained either elusive (one tumour) or could not be satisfactorily confirmed by clinical screening methods (two tumours). *RB1* promoter hypermethylation had previously been excluded in these tumours. Additionally, in one case, tumours from both the left and right eye were available from a paediatric patient with a germline *RB1* mutation providing an opportunity to determine phylogenetic relatedness between bilateral tumours in germline mutation carriers.

## 2. Results

### 2.1. All Retinoblastoma Tumours Harbour At Least One RB1 Mutation

**Figure 1:**
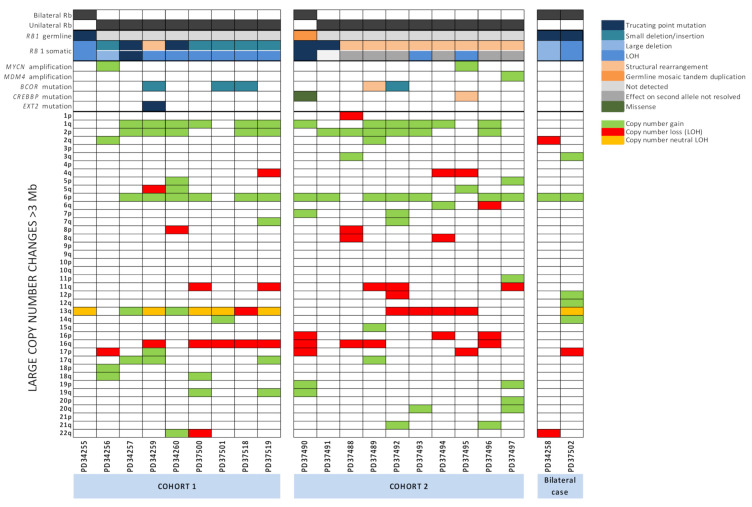
Driver gene mutations and copy number aberrations in retinoblastoma. Summary of *RB1* mutations, mutations in driver genes and large copy number changes >3 Mb in size.

At least one *RB1* mutation was identified in each of the 19 tumours interrogated from Cohorts 1 and 2 (Figure 1, Table S1). We confirmed all the mutations identified by clinical screening in tumours from nine patients of Cohort 1 (Table 1). One sample from a patient with a germline essential splice site mutation in *RB1* (PD34255) had lost the alternative parental allele through loss of heterozygosity (LOH). Of the remaining eight tumours, six had small truncating somatic mutations on one allele (new stop codons, frameshifting indels or essential splice site variants) and lost the other allele through LOH or multiexon deletion (PD34256, PD34260, PD37500, PD37501, PD37518, and PD37519), one case had a large-scale structural deletion and LOH of the alternative allele (PD34259) and one case had two small truncating variants (PD34257), a somatic essential splice site mutation together with a somatic nonsense mutation.

In Cohort 2, of twenty disrupted alleles that could possibly be identified across the ten patients, one was not detected (PD37491) and six could not be definitively resolved. We confirmed four previously discovered *RB1* disruptions (a in Table 1: p.(R255*) in PD37490, p.(R358*) in PD37491, and LOH in PD37493 and PD37495), provided confirmatory evidence for three possible mutations (c in Table 1), provided clarity on mutational mechanism for another three (b in Table 1) and identified three new *RB1* disruptions caused by structural variation (d in Table 1: PD37488, PD37493 and PD37495). 

#### 2.1.1. WGS Reveals Novel *RB1* Disruptions by Structural Variation

Novel *RB1* disruptions were successfully identified in three tumours, two of which had previously characterised LOH of *RB1*. In PD37495, balanced translocations between intron 2 of the *RB1* gene with chromosomes 16 and 18 confirmed disruption of *RB1* and thus biallelic *RB1* loss. In PD37493, biallelic loss was a result of a complex rearrangement involving a translocation between intron 17 of *RB1* and intron 15 of *THSD7B* on chromosome 2. In PD37488, complex intrachromosomal rearrangements resulted in multiple break points transecting *RB1* although the effect on the alternative parental allele could not be resolved.

#### 2.1.2. Diverse Rearrangements Transect *RB1*

**Figure 2:**
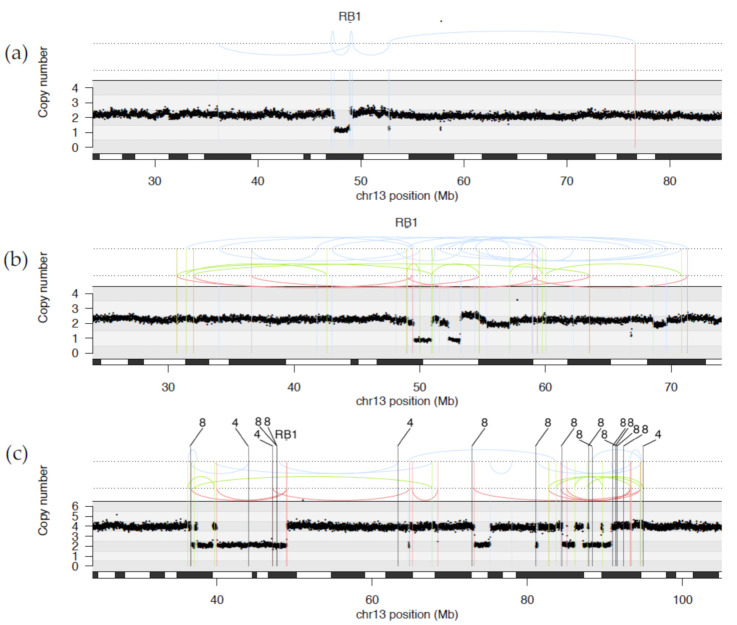
Examples of structural variation affecting the *RB1* locus (**a**) Sample PD37489 demonstrating an intrachromosomal rearrangement, (**b**) Sample PD37488 showing complex intrachromosomal rearrangements, and (**c**) Sample PD37494 showing chromothripsis-like interchromosomal rearrangements between chromosome 13 and chromosomes 8 and 14. Translocation partners for each interchromosomal breakpoint are shown above. Somatic copy number estimates (Y-axis) are plotted against genomic coordinates of chromosome 13 encompassing the RB1 locus (X-axis). Structural variation classes: blue for inversions, red for deletions, green for tandem duplication and black for translocations.

It was previously reported that complex structural variation, specifically a phenomenon called chromothripsis, underpinned some disruptions of *RB1* [19]. Chromothripsis is a compound chromosomal outcome where large numbers of rearrangements are clustered in localised genomic regions, sometimes involving multiple chromosomes, resulting in an oscillating copy number state. In this study, we identified ten tumours with *RB1* structural rearrangements spanning a continuum of complexity (see Figure 2 and Figure S1 for examples of rearrangements disrupting *RB1*). 

Not all structural variations were complex. We observed simple translocations in PD37495 involving a break in intron 2 of *RB1*. The N-terminus of *RB1* was joined to exon 5 of *CREBBP* on chromosome 16, while the C-terminus was translocated to intergenic chromosome 18. Although the LOH of the alternate copy of *RB1* was detected by clinical screening, the translocation break points lie within introns which resulted in a transection of the gene but no discernible change in copy number. Thus, the rearrangement was not detected by customary clinical testing. 

A diverse range of more complex rearrangements were also observed. One relatively complicated rearrangement was detected by clinical testing as a deletion involving exon 2 (PD34259 in cohort1). WGS revealed a more complicated structural outcome where a deletion may have arisen from multiple inversions resulting in loss of the 5′ region of the *RB1* gene including exons 1 and 2 and a separate deletion of other copy of exon 2 (Figure S1).

In three tumours, PD37488, PD37489 and PD37496, multiple rearrangements transected *RB1* as part of a wider collection of complex, intrachromosomal rearrangements (Figure 2a,b). These tumours contained between 7 and 64 break points on chromosome 13, with 3 to 4 break points within *RB1* itself. With such complex rearrangement patterns it was not possible to resolve the order of rearrangements or whether both copies of *RB1* were affected.

Five tumours demonstrated complex interchromosomal rearrangement patterns transecting *RB1* through a variety of structural variations including translocations to other chromosomes (PD37492, PD37493, PD37494, PD37495, and PD37497). PD37492 showed multiple interchromosomal exchange between 13q and a region of chromosome 11, resulting in three separate break points within intron 17 of *RB1* and the loss of one copy of the 3′ end of the gene, including exons 18 to 27. Clinical testing of PD37494 had detected a deletion of the 5′ end of *RB1* encompassing exons 1 to 17. However, WGS was able to clarify that this mutation was part of a complex pattern of rearrangements between chromosomes 13, 4 and 8 with a total of over 40 break points. The oscillating copy number states observed are characteristic of a chromothripsis-like pattern, in keeping with previous reports of chromothripsis in retinoblastoma [19] (Figure 2c). 

#### 2.1.3. WGS Provides Clarification of Ambiguous Clinical Testing Results

Notably, the increased resolution afforded by WGS provided clarification of ambiguous results from clinical testing. Case PD37490 contained a somatic point mutation, c.763C>T p.(R255*) on one allele. Clinical screening using MLPA detected a suspected amplification of exons 12 to 17 of *RB1* in the tumour and possible low-level amplification in the normal blood sample. However, RNA was not available to confirm this result. WGS of the tumour confirmed the presence of a tandem duplication with break points in introns 11 and 17, causing gene disruption. Whilst there was no overt increase in read depth in the normal blood sample, reads spanning the tandem duplication break points were detected in the blood DNA sample, albeit at a reduced fraction compared to the tumour. It is therefore possible that the patient is constitutionally mosaic for the tandem duplication. This patient was the only one of three cases with bilateral tumours, without a confirmed germline mutation and therefore the constitutional mosaic tandem duplication of *RB1* presumably acquired during embryogenesis would be entirely consistent with the clinical picture of bilateral tumours in this patient.

Where structural rearrangements are accompanied by large intragenic deletions of *RB1*, the resulting exonic deletions can be detected by clinical screening methods, as seen in PD34259, PD37489, PD37492 and PD37494. 

In two additional cases, where intragenic *RB1* deletions were suspected by clinical screening but could not be confirmed at the transcript level, WGS provided confirmatory evidence of *RB1* disruption. In PD37497, a monoallelic deletion of exons 23 and 24 was suspected by clinical screening. WGS revealed a complex, interchromosomal rearrangement between chromosomes 13, 7, 8 and 14 including 4 break points in *RB1*. Two of the break points involved in an inversion and tandem duplication, lie in close proximity to the 5′ and 3′ intron/exon boundaries of exons 23 and 24 (72 bp and 49 bp, respectively). In PD37496, a monoallelic deletion of exon 4 was suspected by clinical screening. WGS showed an extremely complex intrachromosomal rearrangement pattern including four break points within the *RB1* gene. Although no changes in copy number were seen, two break points involved in a deletion and inversion lie in close proximity to the 5′ and 3′ intron/exon boundaries of exon 4 (170 pb and 302 bp). In both cases, these rearrangements may have disrupted the binding or orientation of probes used in clinical screening leading to the impression of deleted exons, when the gene was in fact disrupted by alternative structural aberrations.

PD37495 and PD37493, discussed above, had translocations disrupting *RB1* in conjunction with LOH and hence biallelic mutation was confirmed. However, in the six other cases, it was not possible to determine whether both copies of the *RB1* gene were disrupted by rearrangements. WGS can add increased granularity to the details of *RB1* mutations involving structural rearrangements and can provide explanations for suspected intragenic copy number changes detected by standard clinical methods which cannot be confirmed at the transcript level. However, with such complex rearrangement patterns present in some retinoblastoma samples it is not possible to resolve the precise order of rearrangements and whether both copies are affected without also employing alternative approaches such as long read sequencing.

We thus note that nine of ten cases (90%) where biallelic *RB1* mutations were not previously identified had simple rearrangements, complex intrachromosomal as well as complex interchromosomal rearrangements, and one incidence of chromothripsis-like pattern observed. This highlights the contribution of structural variation to the development of retinoblastoma. In contrast to a previous report that 30% of cases (*n* = 3/10) with no detectable *RB1* mutation by exon sequencing had *RB1* disruption via chromothripsis [19], our study indicates that, *RB1* transection can occur through a wide variety of rearrangement mechanisms, and not necessarily via chromothripsis. These results highlight the importance of searching for *RB1* inactivating mutations of all possible types in Rb tumours. 

### 2.2. Retinoblastoma Tumours Harbour a Low Burden of Mutations 

The overall tumour mutational burden of retinoblastoma is low. An average count of 275 (range 26–931) (density 0.085 per Mb), 70 (range 19–231) (density 0.021 per Mb) and 17 (range 1–66) (density 0.005 per Mb) variants were seen for substitutions, small insertions/deletions and structural rearrangements, respectively (See Tables S2–S4 for somatic mutations). Structural variation involved in complex rearrangement patterns leading to disruption of *RB1* in eight tumours contributed to the majority of the total rearrangements (191 of the total 356 rearrangements detected). Tumours with simple point mutations and deletions of *RB1* harboured a lower incidence of structural rearrangements (average 6, range 1–15). Two large-scale WGS studies of multiple paediatric cancer types have reported very low mutation burden in all paediatric cancers [24,25]. Our reported mutation burdens are in keeping with these previous reports. 

### 2.3. Retinoblastomas Are Not Driven by Ultraviolet Damage

Mutational signatures are characteristic patterns of mutations that provide a record of the types of DNA damage that have occurred during the development of a cancer [26]. Due to the very low mutation burden and small sample size, it was not possible to perform de novo mutational signature extraction to identify the mutational signatures present in retinoblastoma. However, we did perform a “fitting” experiment, asking whether mutational signatures that have been previously reported in cancers were present in this dataset [26]. Caution should be exercised when performing signature fitting without prior knowledge of the mutational signatures contributing to a particular cancer type, since overfitting of inappropriate signatures can occur. Grobner et al. attempted to perform signature fitting in only four retinoblastoma WGS as part of a large study of 547 paediatric tumours from 24 cancer types [24]. However, with very small sample numbers, signature contribution estimates are likely to be unreliable.

**Figure 3:**
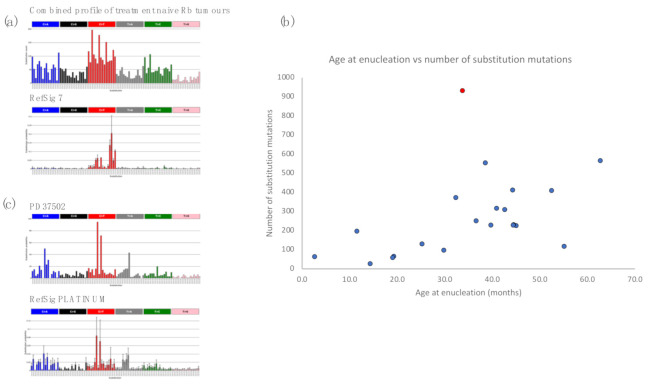
Mutational signatures in retinoblastoma (**a**) 96-trinucleotide mutation profile plots. Single-base pair substitutions are converted to the pyrimidine context and each of the six substitution subtypes, C>A, C>G, C>T, T>A, T>C, and T>G, plotted according to the bases immediately 5′ and 3′ to the mutated base. Profiles produced using Signal (https://signal.mutationalsignatures.com/ (accessed on 10 December 2020)) [33]. Top, profile of the combined substitution mutations from all twenty treatment-naive tumours. Bottom, profile of UV light-associated signature RefSig 7 shows no similarity to that of the retinoblastomas. Reference signature profiles are the mean of tissue specific signatures [33], with error bars representing 1 standard deviation from the mean (https://signal.mutationalsignatures.com/ (accessed on 10 December 2020)). (**b**) Scatter plot showing the correlation between age of enucleation (x axis) and number of substitution mutations (y axis). Blue dots, treatment-naive samples. Red dot, the bilateral tumour exposed to platinum-based chemotherapy (PD37502). (**c**) Top, profile of the bilateral tumour exposed to platinum-based chemotherapy (PD37502). Bottom, profile of the platinum therapy associated mutational signature shows a striking similarity to that of PD37502 (Cosine similarity = 0.96).

Of note, ultraviolet (UV)-related DNA damage has been raised as a potential contributor to the development of retinoblastoma [27], although in a subsequent study, it was suggested that it was a confounding factor [28]. In conjunctival melanoma, an adult eye cancer, a striking contribution of C>T mutations characteristic of UV damage has been demonstrated [29]. By contrast, sequencing studies of another adult eye cancer, uveal melanoma, have not found UV to be a contributory factor to tumorigenesis [30,31]. Signature fitting in the 21 WGS retinoblastomas although limited by the low mutation numbers, showed no evidence of the signature of UV light (Figure 3a), indicating that UV damage is unlikely to be a major source of DNA damage in this tumour type. Furthermore, features such as transcriptional strand bias and double substitutions, which are additional characteristics of UV-associated damage, were also not observed in the retinoblastomas (Tables S5 and S6).

Somatic mutations are continuously acquired at each cell division during the course of human life. The mutational processes that contribute to these replicative errors would thus be correlated to human age [32]. We assessed the correlation between total number of substitution mutations per sample with the age of enucleation. We find a clear correlation between mutation burden and age of enucleation of treatment-naïve tumours (*r*^2^ = 0.835, *p*-value 0.001) (Figure 3b). This is in keeping with mutational processes that are mainly associated with cell division and an absence of other mutational processes.

### 2.4. Insights from Analysis of Independent Tumours in a Bilateral Retinoblastoma Case

A patient with no prior family history of ocular tumours presented aged 19 months with bilateral retinoblastomas, International Classification of Retinoblastoma Group E/E. The right eye had raised intraocular pressure, iris neovascularisation and multiple white calcified retinal tumours, with lens touch, a total retinal detachment and multiple vitreous seeds. The left eye had early iris neovascularisation, with normal intraocular pressure and a large white calcified tumour touching the lens, with multiple subretinal seeds. The right eye was enucleated immediately and an attempt was made to save the left eye as it was slightly less advanced. The patient was treated with 6 cycles of systemic JOE chemotherapy (vincristine (1.5 mg per 10 kg), carboplatin (600 mg per 10 kg), and etoposide (300 mg per 10 kg)) over a four-month period. Despite an initial reduction in tumour size of the left eye, there was evidence of localised relapse at fundoscopy and the remaining left eye was enucleated at 34 months of age.

A germline *RB1* c.607+1G>T splice donor site mutation was identified through clinical screening. This mutation is predicted to cause a frameshift (p.lle181Glyfs*8), which follows skipping of exon 6 [34]. However, diverse somatic mutations of the alternate parental copy of *RB1* were identified in the two tumours (PD34258 (right eye tumour), PD37502 (left eye tumour)).

A large somatic deletion of *RB1* involving exons 8 to 11 was identified in the tumour of the right eye. It also had 65 substitutions, 28 small indels, 8 rearrangements, gain of 6p and deletions of 2q and 22q. In the tumour of the left eye, the remaining copy of *RB1* was lost via a large somatic deletion encompassing most of chromosome 13q, resulting in LOH of *RB1*. A substantially greater number of mutations was detected including 931 substitutions, 103 small indels, 5 rearrangements, gains on chromosomes 3q, 6p, 14q, 12p and 12q and two deletions on chromosomes 17 and X. None of the somatic mutations were shared between the tumours from the two eyes, confirming that the two tumours had arisen independently.

The increased burden of mutation could result in part from an increased age as the second tumour was removed 15 months after the first. However, when assessing mutation burden as a function of age, this tumour was a clear outlier (Figure 3b), suggesting mutational processes in addition to those associated with age were operative in this tumour. Indeed, the substitution mutation profile was consistent with an exposure to platinum, which is a key component of carboplatin (Cosine similarity = 0.96) (Figure 3c). The presence of this signature suggests that this tumour has been derived from an ancestral clone that survived six exposures to carboplatin. Carboplatin inhibits cancer growth by inducing DNA damage and initiating cell death, which has resulted in the acquisition of additional mutations consistent with this exposure and a selection pressure for gene mutations that facilitate survival of that clone, during its resurgent growth prior to enucleation. 

### 2.5. Large-Scale Copy Number Changes 

A number of studies have identified large-scale copy number aberrations (CNAs) (greater than 3 Mb in size) in human retinoblastoma [19,20]. In treatment-naive samples in this study, gains in 1q (12/20, 60%), 2p (11/20, 55%) and 6p (14/20, 70%) and losses in 16q (9/20, 40%) were observed, in keeping with previous reports (Figure 1). Copy number changes in chromosome 13, harbouring *RB1*, included gains, losses and copy number neutral LOH.

Notably, tumours with gains in 1q, 2p and 6p rarely had them as singular events. A singular gain of 1q (1/20, 5%), 2p (1/20, 5%) or 6p (2/20, 10%) were seen in only four individual cases. Paired gains were also infrequent, no examples of 1q+2p were observed, while 2p+6p (1/20, 5%) and 1q+6p (2/20, 10%) were present. In contrast, 45% of the tumours examined had concurrent gains in 1q, 2p and 6p (9/20). This co-occurrence of CNAs may suggest that either the gains observed in 1q, 2p and 6p are very strongly selected for in this tumour type, or that a common mechanism is driving these gains.

### 2.6. RB1 Mutations and N-MYC Dysregulation Are Not Mutually Exclusive and N-MYC Dysregulation Is Universal

It was originally believed that profound amplification of *MYCN* (≥10 copies) was only associated with a small subset of retinoblastomas [35] that lack detectable *RB1* mutations. As a consequence, it was suggested that *MYCN* amplifications and *RB1* mutations were mutually exclusive and that N-MYC dysregulation could provide an alternative pathway to malignancy in retinoblastomas [17]. Subsequently, a separate report indicated that six of eight tumours with *MYCN* amplifications also contained at least one *RB1* mutation [19]. Whilst these driver events are not mutually exclusive, a small proportion of *RB1* wild-type Rb with *MYCN* amplifications may still exist [19]. Here we identified two tumours which were biallelically null for *RB1* that also harboured focal *MYCN* amplifications, in the case of PD34256 an amplification of 168-fold, while PD37495 was amplified 51-fold (Table S7). Both *RB1* mutations were detected by clinical screening in one tumour (PD34256). However, in the other (PD37495), clinical screening only detected LOH across *RB1*, the alternative allele being disrupted by balanced translocations with chromosomes 16 and 18 which were only detected by WGS, thus presenting the possibility that the proportion of *MYCN* amplified Rb tumours perceived to be *RB1* wild type or have monoallelic *RB1* loss maybe smaller than previously thought due to undetected *RB1* mutations involving structural rearrangements. 

Contrary to previous reports [17], we did not see any difference in the age of diagnosis for the two patients with *MYCN* amplification compared to rest of our cohort. The age of diagnosis was 40.7 months for PD34256 and 38.3 months for PD37495, compared with a mean age of diagnosis of retinoblastoma tumours with somatic *RB1* mutations of 34.32 ± 16.42 months, (95% CI 41.88, 26.72).

Moreover, we performed RNA sequencing of five retinoblastoma samples and compared the gene expression of these tumours to normal retinal tissue to find that all cases of retinoblastoma are associated with an increased expression of N-MYC even in the absence of a *MYCN* genomic abnormality. We observed a 366-fold increase in *MYCN* mRNA expression in the tumour with 168 copies of *MYCN* (PD34256) and 11- to 21-fold increase in expression in 4 tumours with 2 or 3 copies. (Table S7). This suggests that N-MYC dysregulation is a common pathway towards retinoblastoma formation.

### 2.7. Other Potential Driver Mutations Identified in Retinoblastoma Tumours

We identified a total of 94 non-synonymous mutations, which were assessed for potential driver mutations. In addition, we interrogated regions of homozygous deletions and amplifications as putative copy number drivers (Figure 1 and Table S8).

Mutations of *BCOR* were identified in 5 out of 21 tumours, an incidence of 24%. Mutations included 4 small indels resulting in frameshift mutations and a large 588 Kb deletion disrupting the 5′UTR. This frequency is in line with previous reports of loss of function mutations in BCOR (10–23%) [18,20,21,22]. Three patients, PD34259, PD37492 and PD37518 were male and since *BCOR* is located on chromosome X p11.4, these mutations were hemizygous in nature. The two remaining patients with *BCOR* mutations were female (PD37501 and PD37489). Although neither demonstrated LOH across the *BCOR* locus, it is possible that the alternative parental allele in these two tumours may be affected by X-inactivation. BCOR is thought to regulate transcription and is an important potential driver mutation in a number of cancers including acute myeloid leukaemia and CYLD cutaneous syndrome [36,37]. A focal amplification of *MDM4*, resulting in 11 copies of the gene, was observed in one patient (PD37497) consistent with a recent report [18]. *MDM4* encodes an E3 ubiquitin ligase that functions as a negative regulator of p53 activity and it has been postulated that the *MDM4* gene on chromosome 1q32 is the potential oncogene driving the frequent gains of chromosome 1q in retinoblastoma [20,38]. We did not identify any focal amplifications of the *OTX2* gene reported previously [19].

Mutations in the Creb-binding protein gene (*CREBBP*) are associated with follicular neoplasia and lymphoblastic leukaemia [39,40] and occasional focal losses and point mutations of the *CREBBP* gene have been recorded in retinoblastoma tumours [19,20]. We recorded two tumours with mutations in *CREBBP*. In PD37495, *CREBBP* was disrupted as a consequence of a translocation between intron 2 of *RB1* and exon 5 of *CREBBP* located on chromosome 16. However, there was no LOH of the remaining wild-type copy. PD37490a contained a missense mutation, c.3029C>T p.(P1010L), which has not been reported previously in cancer. The significance of both of these mutations is unclear. We also identified a solitary tumour (PD34259) containing a c.2113G>T p.(E705*) mutation in *EXT2*. The exostosin glycotransferase 2 gene (*EXT2*) is thought to act as a putative tumour suppressor gene with regard to the development of osteochondroma tumours [41]. However, since there was no LOH of the wild-type allele, the significance of this mutation is unknown.

## 3. Discussion

Early theories of retinoblastoma development established that loss-of-function mutations in both alleles of the *RB1* gene were required to enable its development. However, the failure to identify mutations of both copies of *RB1* in a proportion of Rb tumours led to speculation that other genes may be involved with alternative pathways to malignancy. These theories were further fuelled by the discovery of a subset of Rb tumours with amplification of *MYCN* which initially appeared to be mutually exclusive with *RB1* mutations [17]. Subsequently, Rb tumours with mutation of both *RB1* alleles and also amplification of *MYCN* were discovered [19].

We show that all Rb tumours in our cohort had at least one *RB1* mutation, including three tumours where clinical testing was not able to confirm the existence of *RB1* mutations. We have demonstrated the power of WGS to identify structural rearrangements disrupting *RB1*, a class of mutation which otherwise goes undetected by standard clinical screening approaches. The wide spectrum of rearrangement patterns capable of disrupting *RB1* highlight the need for exhaustive searching for genomic aberrations in Rb tumours. WGS is able to complement the results of clinical screening by confirming the mechanisms of *RB1* loss. 

However, despite the increased resolution of WGS, with such complicated rearrangement patterns, it was not possible to confirm whether both copies of *RB1* were affected by the rearrangements in some tumours. It is possible that long-range sequencing techniques may be able to resolve the full complexity of these rearrangements. 

Seven tumours remained with unconfirmed biallelic *RB1* mutations. Six of the seven involved highly complex rearrangements. While we could not confirm that these affected both alleles, we could not rule out complex structural variation as the mechanism for inactivation of both copies either. Alternatively, additional *RB1* mutations may have gone undetected by both WGS and clinical screening or may exist in as yet unrecognised regulatory regions outside the coding sequence of the *RB1* gene. 

We identified two examples of Rb tumours with both biallelic *RB1* mutations and *MYCN* amplification. In one example, standard clinical screening detected LOH of the *RB1* locus but was not able to detect the translocation disrupting the remaining allele identified by WGS. Alternative mechanisms for RB pathway inactivation such as phosphorylation of pRb have been proposed in *MYCN*-amplified Rb with apparently intact *RB1* [42]. However, our study suggests a more thorough search for structural rearrangements disrupting *RB1* may reveal that the proportion of *MYCN*-amplified Rb which truly retain intact copies of *RB1* is smaller than previously thought. Furthermore, RNAseq data from five Rb tumours suggested that increased N-MYC expression is a common feature of tumours with 2 or 3 copies (11- to 21-fold), compared to normal retina, and demonstrated a dramatic increase in expression in the 185-fold *MYCN* amplified tumour, thus suggesting that *MYCN* expression may be increased in all Rb tumours, further implicating the role of N-MYC in the development of Rb tumours in general. Indeed, it has been shown that proliferation and survival of retinoblastoma cells require expression of N-MYC [43,44]. In a study of 6 retinoblastomas, Ganguly et al. observed a 9-fold increase in *MYCN* expression, in line with the increases seen in the non-amplified tumours in this study [45]. The numbers in both studies are small and further expression studies on larger cohorts of tumours are warranted to confirm whether *MYCN* over expression is a common feature of retinoblastoma. Targeting N-MYC may offer a potential therapeutic strategy for the treatment of retinoblastoma, especially in cases where its copy number is profoundly increased [46].

It has been postulated that biallelic inactivation of *RB1* can result in benign retinomas and that subsequent genetic alterations are required for progression to retinoblastoma [38]. A number of studies have focused on the search for additional mutations that may be involved in driving Rb. We confirmed the presence of recurrent copy number changes previously identified in Rb [20] and the existence of mutations in *BCOR* and *MDM4*. Our study only interrogated 21 Rb tumours and therefore our ability to detect new potential driver mutations is limited. However, WGS allowed us to look at the full compendium of mutations in our cohort of retinoblastomas and suggests that driver mutations beyond *RB1*, *MYCN BCOR* and recurrent copy number changes on 1q, 2p, 6p, and 16q are rare.

The study of mutational signatures in WGS data from large cohorts of adult tumours has provided exciting insights into the mechanisms of DNA damage and repair operating in cancer and a number of potential therapeutic opportunities [47,48]. However, with the limited number of WGS available and the low mutation burden in this rare paediatric cancer, it was not feasible to perform mutational signature analysis to the same depth. Nevertheless, we were able to make some observations about mutational signatures in Rb. In contrast to conjunctival melanoma, we were able to rule out UV light as a source of DNA damage in Rb. In addition, the association of mutation burden with age suggests mutational processes linked to cell division are most likely to be contributing to somatic mutations. Previous studies using comparative genomic hybridisation have shown similar associations between age of enucleation and increased frequency of another class of mutation, large-scale chromosomal aberrations [49,50,51].

Finally, we describe an interesting case of bilateral retinoblastoma where we had the opportunity to study tumours from both eyes. In this germline *RB1* carrier, we showed that loss of the second allele was different in the two tumours and the lack of shared somatic mutations confirmed that the tumours had arisen independently. The tumour from the eye which remained in situ during chemotherapy contained the mutational signature previously associated with platinum-based therapies, suggesting the recurrence of this tumour could be due to the development of a treatment resistant clone. Exploration of other such cases may provide clues to help identify therapeutics or combinations of therapeutics which may be able to reduce the development of resistance.

## 4. Materials and Methods

### 4.1. Patient Details

Peripheral blood and tumour samples were obtained from patients referred to the Retinoblastoma Service and Retinoblastoma Genetic Screening Unit of the Royal London Hospital (Barts Health NHS Trust) for enucleation. All procedures were performed in accordance with the Human Tissue Act 2004. Informed consent was obtained prior to enucleation from all subjects. Ethical approval for the work was obtained from the National Research Ethics Service Committee London—Hampstead (15/LO/0647) under the project titled “Identification of new mechanisms and targets in retinoblastoma: a cohort study using fresh tissue samples for in vitro studies, leading to novel in vivo imaging techniques and treatment strategies” and the Barts Health NHS Trust Institutional Review Board and Moorfields Eye Hospital Ethical Committee under the project entitled “Expression analysis of proteoglycans in the retina” (10/H0106/57-17ETR57). 

### 4.2. Tissue Processing and DNA/RNA Extraction

DNA was extracted from peripheral blood samples as described previously [52]. Retinoblastoma and retinal tissue samples were dissociated by homogenisation and DNA/RNA isolated using a Qiagen All Prep DNA/RNA Mini Kit in accordance with the manufacturer’s instructions. Control retinal tissue samples were obtained from the enucleated eyes of 3 paediatric Rb cases, from an area which was noted to be geographically distant from cancerous tissue and normal in appearance. The pieces of retinal tissue were separated from the choroid layer underneath, placed into RNAlater and immediately frozen.

### 4.3. mRNA Expression Profiling

mRNA expression was compared in 3 control retinas and 5 retinoblastoma tumours. cDNA was generated using KAPA Stranded RNA-Seq kit with RiboErase (HMR) (Roche, Herts, UK). cDNA synthesis was carried out using Illumina TruSeq RNA sample prep kit version 2 (RS-122-2001) in accordance with the manufacturer’s instructions, with the following variations in protocol, 250 ng total RNA was used as a starting material, fragmentation was carried out for 10 min and 12 cycles of PCR were used. Samples were sequenced in a 24 plex pool on a NextSeq 500 (Illumina, Camb, UK) using 43 bp paired-end sequencing and 16 to 20 million read pairs generated per sample. FastQ files have been deposited at NIH Sequence Read Archive, BioProject ID PRJNA693838.

### 4.4. Analysis of mRNAseq Data

Expressional analysis was conducted using DESeq2, where raw data were normalised by scaling followed by linear modelling and outlier removal. Expression of a transcript was considered to be significantly perturbed in retinoblastoma if its statistical significance was less than *p* = 0.05 after a Benjamini–Hochberg multiple testing correction and its expression was increased or decreased 2-fold (Table S9).

### 4.5. Clinical RB1 Mutation Screening

*RB1* mutation screening in a clinical context was performed at the Royal London Hospital as described previously [9]. Briefly, conformation analysis followed by Sanger sequencing of any exon displaying a profile different from wild-type controls was used to screen all exons, splice sites (except splice acceptor for exon 22) and the promoter region, for point mutations and small insertions and deletions. A combination of an in-house Quantitative Fluorescent PCR (QF-PCR) dosage assay and Multiplex Ligation-dependent Probe Amplification (MLPA *RB1*) (SALSA P047 *RB1*) were used to detect large deletions and gains of all exons and splice sites, the promoter region and the 3′Untranslated Region (UTR). Where possible, dosage mutations were confirmed by reverse transcriptase polymerase chain reaction (RT-PCR) and Sanger sequencing of the *RB1* gene transcript from 5′*RB1* to 3′UTR (c.-67 to c.*55). *RB1* promoter hypermethylation was investigated using Methylation Specific PCR of the *RB1* promoter [52]. LOH analysis was performed using intragenic and flanking chromosome 13 polymorphic markers. D13S118, STRs 1 Kb 5′ of *RB1*, RBi.2 (*RB1* intron 2), RBi.4 (*RB1* intron 4), *RB1*.20 (*RB1* intron 20), STR 17 Kb 3′of *RB1*, D13S1307 (all on 13q14.2).

### 4.6. Whole-Genome Sequencing

Whole-genome sequencing was performed using standard methods as previously described [53]. Short-insert 500 bp genomic libraries were constructed in accordance with Illumina library protocols and 150 base paired-end sequencing was performed using an Illumina HiSeq X Ten. Average sequence coverage was 37.6 for both tumour and normal samples. The resultant reads were aligned to the reference human genome (GRCh37) using a Burrows–Wheeler Aligner, BWA (0.7.16a (r1181)). Single-nucleotide substitutions were called using CaVEMan (Cancer Variants through Expectation Maximisation, http://cancerit.github.io/CaVEMan (accessed on 10 December 2020)). Insertions and deletions (indel) were called using split-read mapping using a modified Pindel version 2.0 (http://cancerit.github.io/cgpPindel/ (accessed on 10 December 2020)). Structural rearrangements were identified by grouping discordant read pairs that point to the same break point event using the BRASS (break point via assembly) algorithm, (github.com/cancerit/BRASS) followed by de novo local assembly using Velvet to determine the exact co-ordinates and features of a break point junction sequence. All annotation was to Ensembl build 75. Non-synonymous point mutations and small indels were assessed for potential driver mutations by comparison to the list of genes in the Cancer Gene Census (https://cancer.sanger.ac.uk/census (accessed on 10 December 2020)). Mutations within these genes were considered to be potential drivers if the same mutation exists multiple times in the COSMIC database. In addition, mutations in genes which are reported in the Cancer Gene Census as tumour suppressor genes were considered to be potential drivers if the mutation was predicted to result in a premature truncation (nonsense, essential splice, frameshift mutations). The WGS data have been deposited in the European Genome-phenome Archive (EGA) database under the accession code EGAD00001006431.

### 4.7. ASCAT Copy Number Analysis

Allele-specific copy number analysis of tumours were performed using ASCAT (v2.1.1) applied to whole-genome sequencing data as described previously [53]. ASCAT takes non-neoplastic cellular infiltration and overall tumour ploidy into consideration to generate integer-based allele-specific copy number profiles for the tumour cells. Copy number values and estimates of aberrant tumour cell provided by ASCAT were then put into the CaVeMan substitution algorithm. In addition, ASCAT segmentation profiles were used to establish the presence of LOH across the *RB1* gene and to search for homozygous deletion and amplification of cancer driver genes. Copy number aberrations were considered as amplifications if the copy number was more than or equal to 5 for diploid tumours (with ploidy < 2.7 n) or more than or equal to 9, for tumours with evidence of whole-genome duplication with ASCAT ploidy >2.7 n. Large-scale copy number changes of greater than 3 Mb were considered to be gained if the total copy number exceeded 2 in diploid genomes and 4 in tumours with whole-genome duplication, and losses if the minor allele copy number was 0 indicating regions of LOH. Regions restricted to telomeres and those spanning centromeres where the size of the segment is not reliable were excluded from the large-scale copy number analysis.

### 4.8. Mutational Signature Analysis

Due to the very low mutation burden present in retinoblastomas, any background artefactual noise or contamination with germline SNPs will make up a much higher proportion of mutations than observed in typical adult solid tumours with a much higher mutation burden. Consequently, even the smallest amount of contamination with germline SNPs, either resulting from failure to remove personal SNPs by comparison with the matched normal or from contamination with DNA from another individual, will make a significant difference to mutation burden counts and mutational signatures analysis. Therefore, after seeking potenital driver mutations, we performed a stringent filtering process on single-nucleotide variant (SNV) substitution mutations to increase the level of high-confidence somatic mutations for subsequent analysis. This additional filtering involved removal of all SNVs present in the 1000 genomes project, as indicated by population frequency in 1000G obtained from dbSNP. In addition, all SNVs with a variant allele fraction (VAF) less than 0.2 were also removed. The resulting high-confidence SNVs were used to investigate the correlation of mutation burden with age and for mutational signature analysis.

To investigate the contributions of substitution signatures, we used a fitting approach as described by Degasperi et al. [33]. Briefly, the substitution profile is described as a 96-channel vector. For each mutation, of which there are six substitution classes of C>A, C >G, C>T, T>A, T>C, and T>G, the flanking 5′ and 3′ sequence context is taken into account giving a total of 96 channels. A given set of mutational signatures were fitted into the mutational profile of each sample to estimate the exposure of each of the given signatures in that sample. The fitting algorithm detects the presence of mutational signatures with confidence, using a bootstrap approach to calculate the empirical probability of an exposure to be larger or equal to a given threshold (i.e., 5% of mutations of a sample).

## 5. Conclusions

We have used WGS to comprehensively investigate the mutations driving tumorigenesis in a cohort of 20 sporadic retinoblastomas. WGS has revealed the wide range of structural rearrangements capable of disrupting *RB1* which may otherwise go undetected by standard clinical screening approaches. Mutation of additional driver genes beyond *MYCN* and *BCOR* are rare. Using WGS, we looked at mutational signatures in Rb and found no evidence for a role of UV light exposure. However, in a patient treated with platinum-based chemotherapy, we were able to demonstrate a contribution from a treatment-related mutational signature. 

Establishing the existence of *RB1* mutations and determining whether they are somatic or germline have huge implications for patients and their families. Confirming that biallelic *RB1* mutations are somatic, resulting in the consequential reduction in risk of bilateral tumours, has the benefit of reducing the frequency of future screening required for the patient and the reassurance of low risk to their relatives, while confirming the exact germline mutation present can aid detection of mutation carriers in other family members. Standard clinical screening techniques are able to identify the majority of *RB1* mutations. However, in those cases where *RB1* mutations remain undetected, extensive search using WGS may help provide the answers which are so vital to the patient. 

## Figures and Tables

**Table 1:** All 21 retinoblastoma tumours examined harbour at least one *RB1* mutation.

*RB1* Mutations		
	Cohort 1: Tumours with Biallelic Mutations Previously Identified	
Tumour	*RB1* mutation allele 1	*RB1* mutation allele 2
PD34255	Germline c.1421+1G>C ESS splice ^a^	LOH ^a^
PD34256	Somatic c.1420_1421+30del32 ^a^	Somatic deletion exons 18 to 27 ^a^
PD34257	Somatic c.1421+2T>A ESS splice ^a^	Somatic c.1333C>T p. (R445 *) ^a^
PD34259	Somatic Rearrangement resulting in deletion exon 2 ^a^	LOH ^a^
PD34260	Somatic c.1363C>T p.(R455*) ^a^	LOH ^a^
PD37500	Somatic c.266_229delTTAAp.(T77fs*33) ^a^	LOH ^a^
PD37501	Somatic c.393_396dupCTTT p.(N133fs*2) ^a^	LOH ^a^
PD37518	Somatic c.1389dupA p.(E464fs*11) ^a^	LOH ^a^
PD37519	Somatic c.173delC p.(T58fs*7) ^a^	LOH ^a^
	**Cohort 2: Tumours with One or Zero Previously Identified Mutations**	
PD37490	Somatic c.763C>T p.(R255*) ^a^	Germline mosaic duplication of exons 12–17 ^c^
PD37491	Somatic c.1072C>T p.(R358*) ^a^	Not detected
PD37488	Complex intrachromosomal rearrangements, 4 break points in *RB1*.^d^	Effect on second allele could not be resolved
PD37489	Complex intrachromosomal rearrangement, 3 break points in *RB1*. One copy lost due to deletion of exons 1–6. ^b^	Effect on second allele could not be resolved
PD37492	Complex interchromosomal rearrangements, 3 break points in *RB1*. One copy lost due to deletion of exons 18 to 27. ^b^	Effect on second allele could not be resolved
PD37493	Complex interchromosomal rearrangements. *RB1* disrupted by a translocation to chromosome 2. ^d^	LOH ^a^
PD37494	Complex interchromosomal rearrangement, 2 break points in *RB1*. One copy lost due to deletion of exons 1 to 17. ^b^	Effect on second allele could not be resolved
PD37495	*RB1* disrupted by balanced translocations from intron 2 to chromosomes 16 and 18. ^d^	LOH ^a^
PD37496	Complex intrachromosomal rearrangement, 4 break points in *RB1*. ^c^	Effect on second allele could not be resolved
PD37497	Complex interchromosomal rearrangement, 4 break points in *RB1*. ^c^	Effect on second allele could not be resolved
	**Bilateral Tumours from a Single Patient**	
PD34258	Germline c.607+1G>T ESS splice ^a^	Somatic deletion exons 8 to 11
PD37502	Germline c.607+1G>T ESS splice ^a^	LOH

Cohort 1 consists of nine tumours where two clear mutations in *RB1* were identified through clinical screening. In Cohort 2, containing ten tumours, one *RB1* mutation had been previously identified in seven tumours, and in a further three tumours, *RB1* mutations were undetectable (PD37488) or could not be confirmed by clinical genetic screening (PD37496 and PD37497). *RB1* mutations identified by clinical screening and confirmed by WGS are indicated by ^a^, and *RB1* mutations where WGS was able to provide additional information on the mechanism of exon deletions are indicated by ^b^. ^c^ indicates samples where a deletion or gain of exons in *RB1* was suspected but not confirmed by clinical screening and WGS was able to provide an explanation. New *RB1* mutations detected by WGS are indicated by ^d^. Where two tumours from a patient with bilateral tumours were available, WGS confirmed the germline *RB1* mutation in both tumours and identified somatic mutations in the alternative parental allele resulting in *RB1* nullness. Annotation according to CCDS31973.1 (NM_000321.3).

## Data Availability

The WGS data have been deposited in the European Genome-phenome Archive (EGA) database under the accession code EGAD00001006431. RNASeq FastQ files have been deposited at NIH Sequence Read Archive, BioProject ID PRJNA693838.

## Supplementary Materials

The following are available online at https://www.mdpi.com/2072-6694/13/4/754/s1, Figure S1: Examples of structural rearrangements disrupting *RB1*, Table S1: Mutations in *RB1,* Table S2: High-quality substitution mutations (all SNVs present in 1000 genomes project and with VAF in tumour less than 0.2 removed), Table S3: Small insertion and deletion mutations, Table S4: Structural rearrangement mutations, Table S5: Proportion of dinuleotide substitutions, Table S6: Estimate of transcriptional strand bias for substitution mutations, Table S7: (a). The relationship between MYCN copy number and mRNA expression in five retinoblastoma tumours. (b) MYCN copy number for all 21 tumours, Table S8: Potential driver mutations in genes other than *RB1* and MYCN, Table S9: Information relating to RNAseq comparison of three control “normal” retinas and material obtained from five retinoblastoma tumours.
