# Supplementary material for: Whole-Genome Sequencing of Retinoblastoma Reveals the Diversity of Rearrangements Disrupting RB1 and Uncovers a Treatment-Related Mutational Signature
Source: Cancers (Basel). 2021 Feb 11;13(4):754. doi: 10.3390/cancers13040754 (PMC7918943; doi:10.3390/cancers13040754)
Supplement: Supplementary file 1 [file cancers-13-00754-s001.zip › cancers-1056752 - supple/SupplementaryFigure1.pdf]

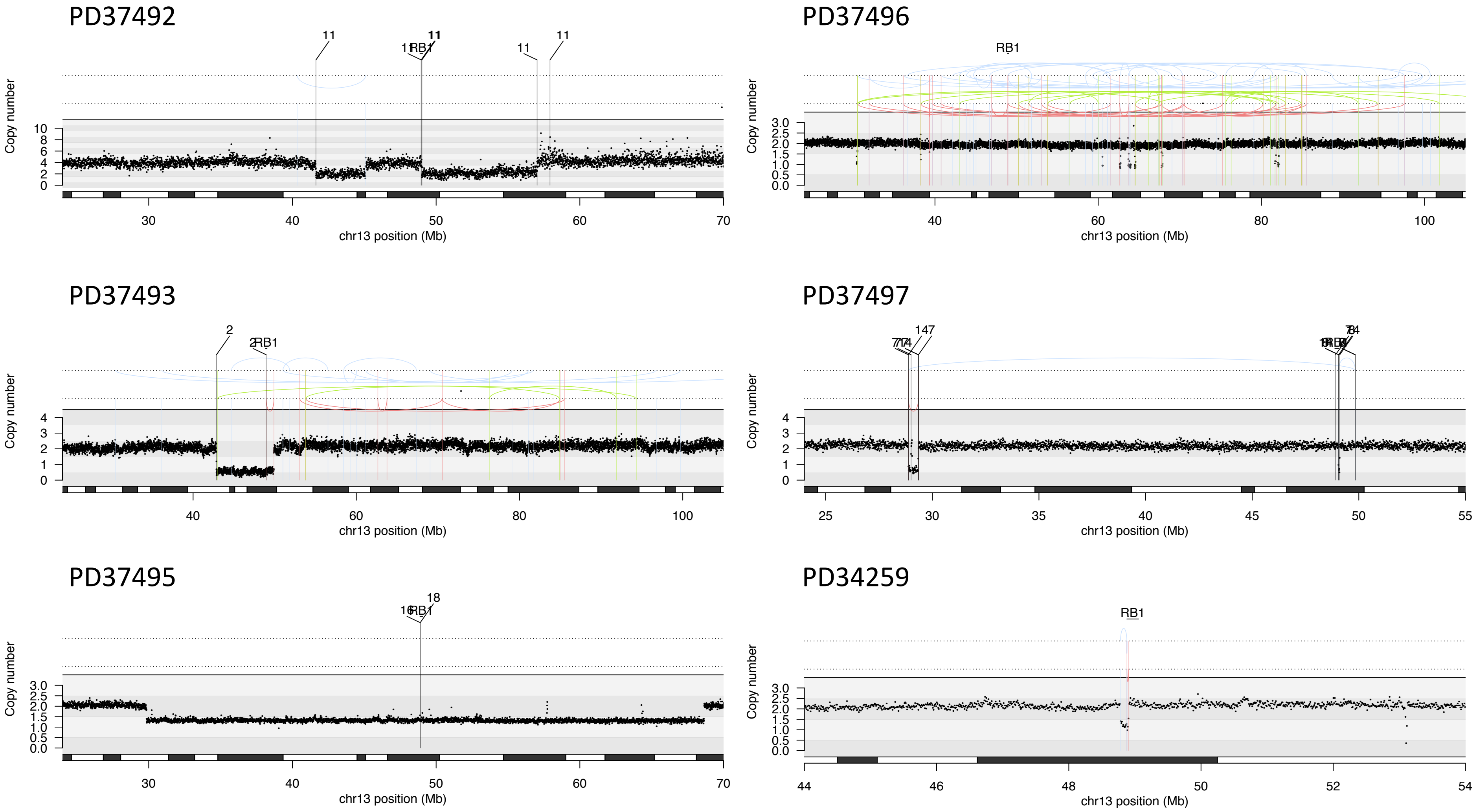

Supplementary Figure 1: Examples of structural rearrangements disrupting *RB1*

Somatic copy number estimates (black dots, Y-axis) are plotted against genomic coordinates of chromosome 13 encompassing the RB1 locus (X-axis). Structural rearrangement types coloured blue for inversions, red for deletions, green for tandem duplication and black for translocations. Chromosome numbering of translocation partners are shown above the plots.
